# Automated pixel-based quantification of porcine circovirus 2 genome in formalin-fixed, paraffin-embedded tissues using *in situ* hybridisation

**DOI:** 10.3389/fvets.2025.1609897

**Published:** 2025-08-20

**Authors:** Mònica Sagrera, Àlex Cobos, Laura Garza-Moreno, Mónica Pérez, Gema García-Buendía, Eva Huerta, Anna Maria Llorens, David Espigares, Marina Sibila, Joaquim Segalés

**Affiliations:** ^1^IRTA, Animal Health, Centre de Recerca en Sanitat Animal (CReSA), Campus de la Universitat Autònoma de Barcelona (UAB), Bellaterra, Spain; ^2^Unitat mixta d’investigació IRTA-UAB en Sanitat Animal, Centre de Recerca en Sanitat Animal (CReSA), Campus de la Universitat Autònoma de Barcelona (UAB), Bellaterra, Spain; ^3^Ceva Salud Animal, Avenida Diagonal, Barcelona, Spain; ^4^WOAH Collaborating Center for Research and Control of Emerging and Re-emerging Pig Diseases (IRTA-CReSA), Barcelona, Spain; ^5^Departament de Sanitat i Anatomia Animals, Facultat de Veterinària, UAB, Barcelona, Spain

**Keywords:** porcine circovirus 2, *in situ* hybridisation, qPCR, RNAScope^®^, digital pathology

## Abstract

**Introduction:**

Detection of porcine circovirus 2 (PCV2) in lymphoid tissues is essential for diagnostic and research purposes. *In situ* hybridisation (ISH) enables the localisation of viral genomes in tissue sections but is traditionally assessed visually, which may introduce subjectivity.

**Methods:**

This study developed an automated pixel classifier to quantify the PCV2 genome using RNAscope^®^ ISH technology. Four lymphoid tissues (tonsils and tracheobronchial, mesenteric, and superficial inguinal lymph nodes) from 66 experimentally inoculated pigs were analysed. PCV2 labelling was assessed both visually (scores 0–3) and digitally (percentage of labelled area).

**Results:**

A strong correlation was observed between visual and digital ISH scoring (*ρ* = 0.96), allowing the definition of digital thresholds corresponding to visual scores. Among all tissues, TBLN exhibited the highest PCV2 labelling. This tissue was further evaluated by PCV2 quantitative polymerase chain reaction (qPCR), showing a high correlation with digital ISH results (*ρ* = 0.85).

**Discussion:**

These findings demonstrate the reliability of digital pathology tools for objective quantification of PCV2 in lymphoid tissues. Automated scoring enhances consistency, reduces observer bias, and improves diagnostic efficiency in PCV2 research and surveillance.

## 1 Introduction

Porcine circovirus 2 (PCV2) is one of the most widespread pathogens affecting swine populations worldwide, contributing to a range of clinical manifestations collectively known as porcine circovirus diseases (PCVD) (1). Since its emergence in association with the disease in the late 1990s, PCV2 has become a must-control infectious agent in nearly all commercial pig-producing regions (2). However, PCV2 has been endemic in pig populations since at least the 1960s (3). In addition, wild boar populations are also endemically infected with this virus, harbouring genotypes closely related to those in domestic swine (4, 5).

Porcine circovirus 2 is a small, non-enveloped, single-stranded DNA virus belonging to the family *Circoviridae* (6). The virus causes systemic infections, primarily targeting lymphoid tissues, including tonsils, lymph nodes, and spleen (7, 8). Lymphoid depletion (LD) and histiocytic replacement (HR) are the primary histopathological features associated with PCV2 infection, serving as the diagnostic hallmarks of PCV2 systemic disease (PCV2-SD) (9). Diseased animals experience immunosuppression, making PCV2-SD-affected pigs more susceptible to secondary infections (7). However, PCV2 is not restricted to the immune system, as viral replication has also been observed in the lungs, liver, kidneys, heart, gastrointestinal tract, and reproductive tissues (8, 10–18). Diagnostic confirmation of PCV2-SD generally relies on a three-step approach: (1) the presence of clinical signs and a compatible herd history, (2) the identification of moderate to severe histological lesions in target organs, and (3) the detection of moderate to high amounts of PCV2 within those tissues (19–21).

Detection of PCV2 within tissues is commonly performed using immunohistochemistry (IHC) and conventional *in situ* hybridisation (C-ISH) to identify PCV2 antigens and genome, respectively (22, 23). More recently, RNAscope^®^ technology has been used for research purposes, offering higher sensitivity by detecting individual copies of single-stranded nucleic acids as distinct red dots in tissue sections (24). Additionally, digital pathology tools such as QuPath^®^ (25) have improved the accuracy and reproducibility of viral quantification by enabling automated image analysis of IHC-stained sections (26).

The present study aimed to evaluate a newly developed automated pixel classifier specifically designed for lymphoid tissue samples from experimentally infected animals using PCV2 ISH RNAscope^®^ technology, by correlating its results with visual scoring. Furthermore, the correlation between both visual and digital scores and the PCV2 load in tissue, quantified by real-time quantitative (quantitative polymerase chain reaction [qPCR]), was also analysed.

## 2 Materials and methods

### 2.1 Experimental study design and sample collection

A total of 66 8-week-old pigs were intranasally challenged with 3 mL of inoculum (1.5 mL per nostril) containing 10^4.73^ tissue culture infectious dose 50 (TCID₅₀)/mL of the PCV2b isolate strain Sp-6-11-49-16 (GenBank accession number: EF647673.1). These animals were euthanised (study approval by Ethics Commission of the *Generalitat de Catalunya* through A. M. Animalia Bianya S. L under the reference 028/23) 3 weeks post-challenge, and from each animal, one sample of tonsils (TO) and tracheobronchial (TBLN), mesenteric (MSLN), and inguinal superficial (ISLN) lymph nodes was collected (being a total of 264 samples). All tissue samples were fixed by immersion in 10% buffered formalin, dehydrated, and embedded in paraffin blocks, with all four tissue types from each animal contained within the same block. Fresh samples were also taken separately from each lymph node and stored at −80°C for subsequent PCV2 load investigations by qPCR, selecting the lymph node with the most extensive ISH labelling.

### 2.2 Histopathology and *in situ* hybridisation

From each paraffin block containing the four lymphoid tissues, 4-μm-thick sections were cut, stained with hematoxylin–eosin (HE), and used for examining the presence of lesions indicative of PCV2 infection, such as LD and HR. Another section of each block was prepared for detection of the PCV2 genome using ISH RNAscope^®^ Technology (ACDBio, Newark, California, USA), according to manufacturer procedures and as previously described (27). A PCV2-positive control tissue (containing a high amount of viral antigen detected by IHC) was also included to validate the ISH assay. Briefly, LD, HR, and the amount of PCV2 antigen were visually scored from 0 (no lesions or no staining) to 3 (severe lesions or widespread antigen distribution) (27). Moreover, digitalisation of the ISH sections was performed using the MoticEasyScan One^®^ system (Motic, Hong Kong, China). The amount of PCV2-labelled area was analysed using QuPath^®^ 0.5.1. software (25). A pixel classifier was trained using QuPath^®^ to quantify the labelled area within tissues using a random tree classifier and very high resolution (0.26 μm/px). The trained classifier was then used to measure labelling in all lymph nodes and TO after manually delimiting the lymphoid tissue and excluding the adjacent tissue. The labelled area was recorded as a percentage of the area (labelled area/total area). Finally, all sections analysed by the pixel classifier were manually reviewed to identify potential preparation artifacts. If artefacts were detected, the sample was reanalysed with the appropriate filtering adjustments.

### 2.3 DNA extraction and detection of PCV2 by qPCR

DNA was extracted from 200 μL of the supernatant of macerated TBLN using the MagMAX^®^ Pathogen RNA/DNA Kit (Applied Biosystems, Waltham, Massachusetts, USA), following the manufacturer’s protocol. Negative controls were included in each extraction to ensure that no contamination occurred. To detect and quantify PCV2, the LSI VetMAX™ Porcine Circovirus Type 2 Quantification qPCR assay kit (Thermo Fisher Scientific, Waltham, Massachusetts, USA) was employed. Each qPCR plate contained a standard curve, negative controls, and an internal positive control (IPC) for quality assurance. The limit of quantification (LOQ) was 1.0 × 10^4^ DNA copies/mL, and the limit of detection (LOD) was 4 × 10^3^ DNA copies/mL for tissue supernatant (28). The qPCR results were log_10_ transformed and categorised into three groups: below LOD (<3.6 log_10_), positive but not quantifiable (3.6–4.0 log_10_), and positive and quantifiable (>4.0 log_10_), as previously described (28). For statistical analysis, the following assumptions were made based on the mentioned previous study (28): undetermined or below the LOD values were assigned a value equal to half of the LOD (which corresponds to 3.3 log₁₀). At the same time, positive but non-quantifiable results were set to the LOQ value (4.0 log₁₀).

### 2.4 Statistical analyses

The statistical analyses were performed using GraphPad^®^ (La Jolla, CA, USA) and R Studio^®^ (Boston, MA, USA). The normal distribution of the studied variables (histopathology LD and HR results, visual score, digital score, and PCV2 log_10_ load in tissue supernatant) was assessed using the Shapiro–Wilk test, and correlations between variables were evaluated using Spearman’s rank correlation test. The percentage of each tissue classified within each visual and digital score category was compared using Fisher’s exact test.

## 3 Results

None of the animals exhibited clinical signs compatible with PCV2-SD during the study, and no significant findings were observed at necropsy. Histopathological analysis revealed that only 7.6% of animals (5/66) had LD and/or HR lesions. Two of them had an HR score of 1 in the TBLN, while another two pigs showed the same score of 1 in 3 different lymph nodes (TBLN, MSLN, and ISLN). The fifth animal had a more extensive involvement, with an HR score of 2 and an LD score of 1 in the TBLN and ISLN, as well as an HR score of 1 in the MSLN and TO.

**Figure 1:**
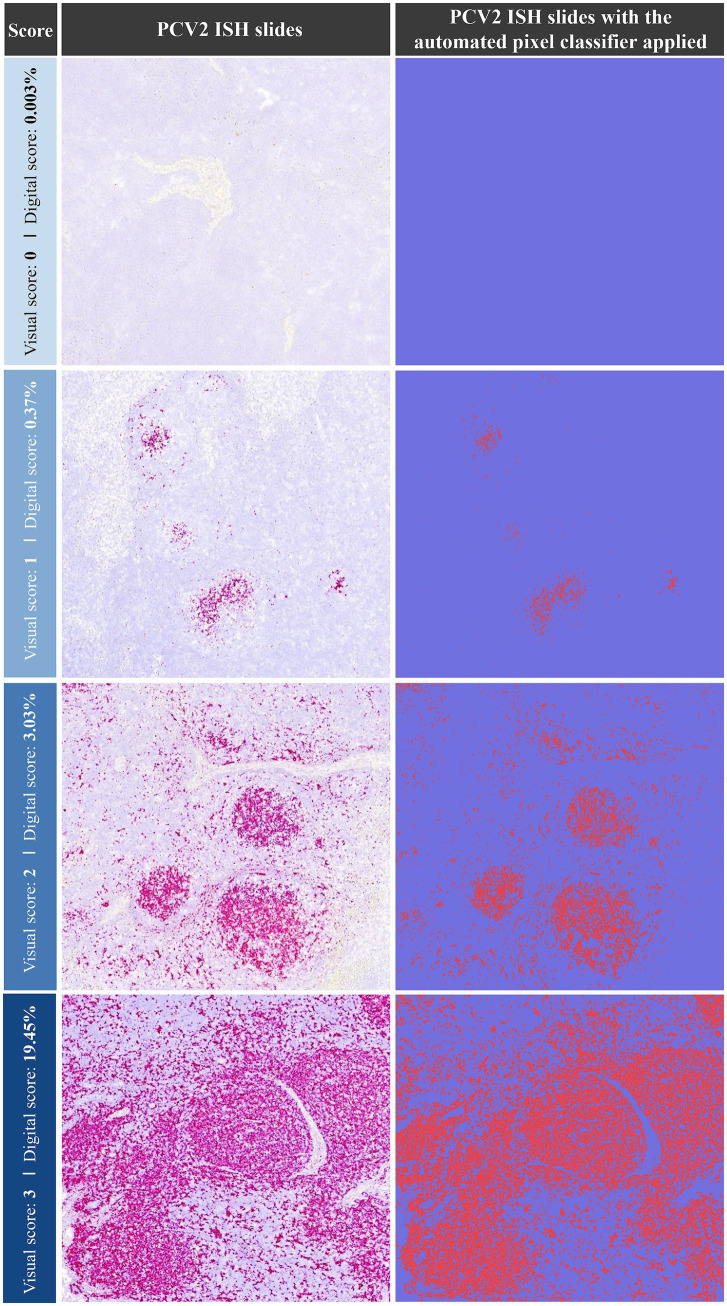
Representative images of PCV2 ISH staining (left column) and the corresponding automated pixel classification analysis (right column). Examples of ISH visual scores (0–3) are shown alongside their respective digital scores (% of labelled area). Each pink dot (left) and red dot (right) represents a PCV2 genomic copy. ISH: *in situ* hybridisation; PCV2: porcine circovirus 2.

The visual scoring system ISH identified PCV2 labelling in 34.8% (23/66) of TO, 42.4% of TBLN (28/66), 33.3% of MSLN (22/66), and 28.8% of ISLN (19/66) samples (Table 1). Based on these findings, the following thresholds for the percentage of labelled area (digitally quantified) corresponding to the visual scores were established as follows: ≤0.0025% (score 0), 0.0026–1.0% (score 1), 1.01–5.0% (score 2), and >5.0% (score 3). A representative image for each of the score values of PCV2 ISH is provided in Figure 1. The automated pixel classifier detected PCV2 in 34.8% (23/66) of TO, 42.4% of TBLN (28/66), 31.8% of MSLN (21/66), and 30.3% of ISLN (20/66) samples (Table 1). The comparison between the digital and visual PCV2 ISH scores across all tissues revealed a strong and statistically significant Spearman’s correlation (*ρ* = 0.96) (*p* < 0.05). Conversely, when both scores were correlated with LD and HR lesion scoring obtained through histopathological analysis across all tissues, lower correlations were observed for both digital and visual scores with LD (*ρ* = 0.16) and HR (*ρ* = 0.38), which were statistically significant (*p* < 0.05).

**Table 1:** Percentage and number of animals per lymphoid tissue (TO, TBLN, MSLN, and ISLN), visual (scored 0–3) and digital (percentage of labelled area) scores obtained from the PCV2 *in situ* hybridisation.

Lymphoid tissue	Visual score (0–3)				Digital score (labelled area, %)			
	0	1	2	3	≤0.0025%	0.0026–1.00%	1.00–5.00%	>5.00%
TO	65.2% (43/66)	24.3% (16/66)	6.1%^a^ (4/66)	4.5% (3/66)	65.2% (43/66)	25.8% (17/66)	4.5%^a^ (3/66)	4.5% (3/66)
TBLN	57.6% (38/66)	15.1% (10/66)	19.7%^b^ (13/66)	7.6% (5/66)	57.6% (38/66)	15.1% (10/66)	19.7%^b^ (13/66)	7.6% (5/66)
MSLN	66.7% (44/66)	21.2% (14/66)	7.6%^a,b^ (5/66)	4.5% (3/66)	68.2% (45/66)	19.7% (13/66)	6.1%^a^ (4/66)	6.1% (4/66)
ISLN	71.2% (47/66)	18.2% (12/66)	7.6%^a,b^ (5/66)	3.0% (2/66)	69.7% (46/66)	19.7% (13/66)	7.6%^a,b^ (5/66)	3.0% (2/66)

TO, tonsil; TBLN, tracheobronchial lymph node; MSLN, mesenteric lymph node; ISLN, inguinal superficial lymph node; PCV2, porcine circovirus 2. Different superscript letters indicate statistically significant differences between lymph nodes for each score value (*p* < 0.05).

**Figure 2:**
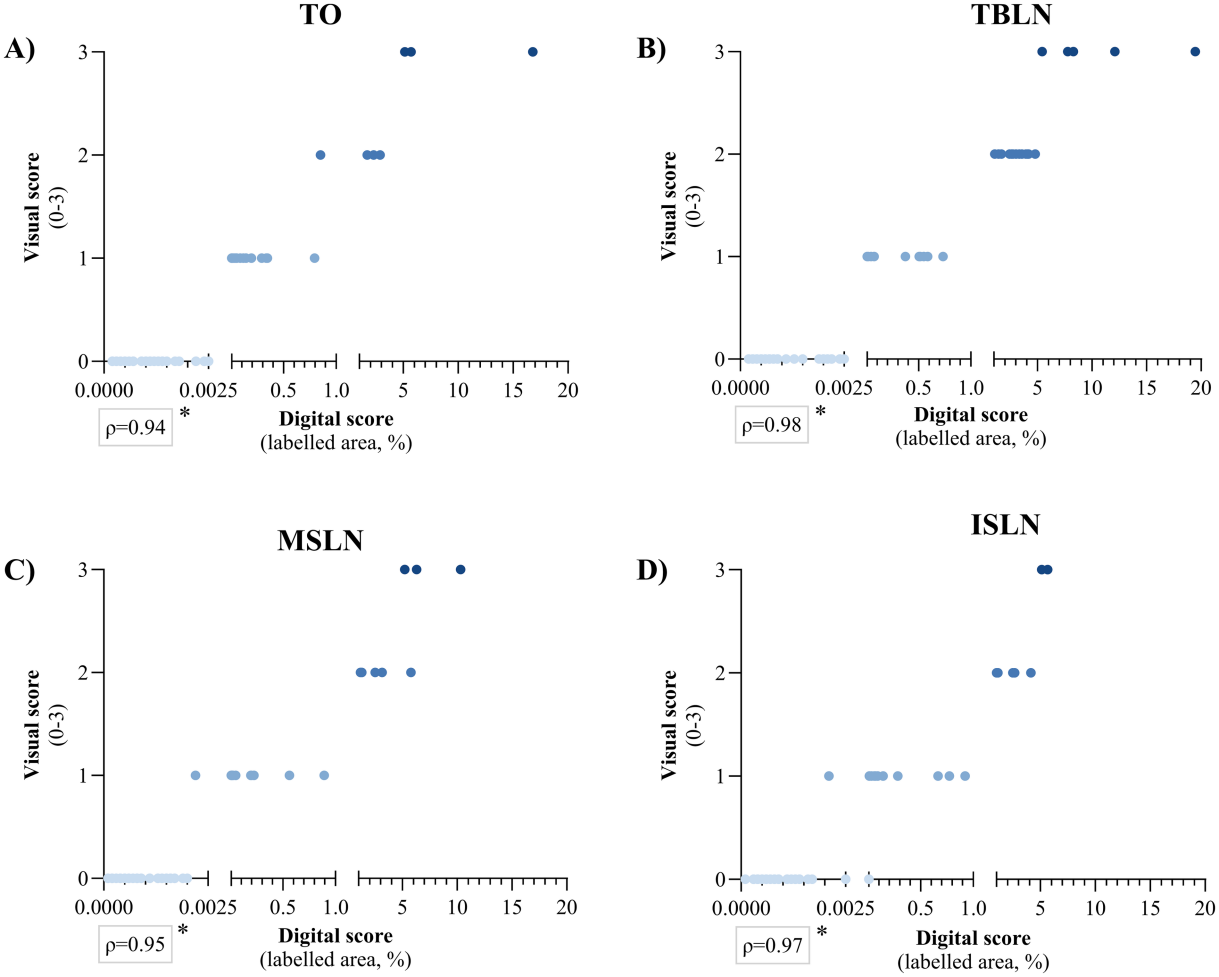
Spearman’s rank correlation between PCV2 *in situ* hybridisation visual score (0–3) and digital score (labelled area expressed in percentage) for **(A)** tonsil (TO), **(B)** tracheobronchial lymph node (TBLN), **(C)** mesenteric lymph node (MSLN), and **(D)** inguinal superficial lymph node (ISLN). A single asterisk (*) indicates that Spearman’s correlation coefficient is statistically significant (*p* < 0.05). The colour of the points corresponds to the visual score, ranging from light blue (0) to dark blue (3), to facilitate the visual interpretation of the graph. PCV2: porcine circovirus 2.

When digital and visual scoring were analysed separately for each tissue, the highest correlation was observed with TBLN (*ρ* = 0.98), while the lowest correlation was observed with TO (*ρ* = 0.94), and this difference was statistically significant in all cases (*p* < 0.05) (Figure 2). Moreover, in both scoring systems, TBLN had a significantly higher number of animals with a visual score of 2 in TO (*p* < 0.05), as well as in MSLN and TO for a digital score of 1.00–5.00% (*p* < 0.05) (Table 1). Given these results, TBLN was the tissue of choice to be processed by qPCR.

When TBLN viral load was assessed with PCV2 qPCR, 74.2% (49/66) were positive. Out of these 49 qPCR-positive samples, 28 showed positive results for both ISH and qPCR (Table 2). The remaining 21 samples that were above the LOQ were classified as score 0 by the visual scoring of ISH and showed values between 0.0 and 0.0025% by the ISH digital scoring, and these samples corresponded mostly to the lowest viral loads within the positive range (Supplementary File 1). Finally, a Spearman’s correlation analysis between PCV2 qPCR results and both ISH visual and digital scoring classification revealed a strong and statistically significant correlation among the three techniques (*ρ* = 0.85) (*p* < 0.05).

**Table 2:** Distribution of PCV2 qPCR results in TBLN according to both visual and digital ISH scores.

TBLN ISH score			TBLN PCV2 qPCR			
Visual score (0–3)	Digital score (Labelled area, %)	Samples (%, *n*)	Samples (%, *n*)			Viral load (log₁₀ PCV2 DNA copies/mL)
			Below LOD	LOD - LOQ	Above LOQ	Mean ± SD (Min–Max)
0	≤0.0025%	57.6% (38/66)	10.6% (7/66)	15.2% (10/66)	31.8% (21/66)	4.6 ± 1.2 (3.3–7.9)
1	0.0026–1.00%	15.1% (10/66)	0.0% (0/66)	0.0% (0/66)	15.1% (10/66)	7.6 ± 1.7 (5.3–10.7)
2	1.00–5.00%	19.7% (13/66)	0.0% (0/66)	0.0% (0/66)	19.7% (13/66)	9.6 ± 0.9 (8.5–11.2)
3	>5.00%	7.6% (5/66)	0.0% (0/66)	0.0% (0/66)	7.6% (5/66)	10.3 ± 0.8 (8.9–11.2)
Total			10.6% (7/66)	15.2% (10/66)	74.2% (49/66)	6.5 ± 2.6 (3.3–11.2)

ISH: *in situ* hybridisation; LOD, limit of detection; LOQ, limit of quantification; PCV2, porcine circovirus 2; TBLN, tracheobronchial lymph node. The proportion of samples (*n*, %) falling below the qPCR LOD (3.3 log₁₀ PCV2 DNA copies/mL), between LOD and LOQ (3.3–4.0 log₁₀), and above the LOQ (>4.0 log₁₀) is displayed. Mean viral load values (log₁₀ PCV2 DNA copies/mL) and corresponding ranges (min–max) are provided for each ISH scoring system.

## 4 Discussion

Since PCV2-SD diagnostics rely on clinical signs, histopathological lymphoid lesions, and detection of the virus in these damaged tissues, the present study developed and evaluated an automated pixel classifier for quantifying PCV2 in lymphoid tissues using ISH RNAscope^®^ technology. Moreover, such automated measures were correlated with the traditional visual scoring (23), LD and HR histopathology lesion scoring, and qPCR-based viral load (29) quantification (in TBLN) in PCV2 experimentally infected pigs.

The automated pixel classifier showed a very strong concordance with visual scoring across all lymphoid tissues, suggesting that digital quantification may eventually replace optical evaluation with enhanced objectivity and reproducibility. These findings align with previous research that used digital quantification of IHC results in animals with different degrees of LD and HR (26). Notably, in the present study, ISH RNAscope^®^ detected PCV2 loads as low as 5.3 log_10_ DNA copies/mL of lymph node supernatant as positive (score 1), outperforming C-ISH and IHC techniques, which typically require higher viral loads (two to three more log_10_ DNA copies) for reliable detection (29, 30). The results are consistent with a recently published study that also reported the superior sensitivity of ISH RNAscope^®^ to detect PCV2 when compared with C-ISH and IHC in porcine dermatitis nephropathy syndrome cases (27), a PCVD that typically displays lower viral loads compared to PCV2-SD.

Notably, the histopathological evaluation in this study confirmed that LD and HR were observed only in a subset of animals. Furthermore, no animal exhibited clinical signs compatible with PCV2-SD throughout the study, highlighting the subclinical nature of this experimental infection. These findings reinforce the relevance of using highly sensitive molecular techniques, such as ISH RNAscope^®^, for detecting PCV2 in subclinical infections, where traditional histopathological approaches may be insufficient. This is demonstrated in the present study by the low Spearman’s correlation coefficients observed between histopathological LD and HR lesion scores and both digital and visual ISH scores, as well as by the lower number of animals with PCV2-compatible lesions detected through histopathology compared to those identified using ISH RNAscope^®^.

A key aspect of the present study was the selection of the TBLN for viral load assessment, given its role as a primary site for PCV2 replication (20, 23). Under the present experimental conditions, TBLN consistently exhibited higher viral loads compared to other lymph nodes, probably due to the nature of the inoculation route, which was intranasal.

Although ISH RNAscope^®^ provides a highly sensitive method for PCV2 detection, its cost remains a limiting factor for routine diagnostic applications. However, its potential for research purposes is advisable, allowing for detailed characterisation of PCV2 distribution within lymphoid tissues. Future studies should explore the applicability of this technique across different PCVD presentations and its potential for detecting early stages of infection before significant histopathological lesions develop, as well as other subclinical presentations (i.e., PCV2-reproductive disease). Such improved sensitivity could provide a deeper understanding of the disease and its dissemination through the organism.

## 5 Conclusion

The automated pixel classifier demonstrated robust performance in quantifying PCV2 in lymphoid tissues, with strong correlations to both traditional visual scoring and qPCR quantification (in TBLN). By reducing operator-dependent variability and facilitating the assessment of ISH results, this approach enables a more objective and efficient evaluation of viral distribution within lymphoid tissues. These findings support the integration of digital pathology tools into PCV2 research, enhancing the accuracy and reproducibility of viral detection methodologies.

## Data Availability

The original contributions presented in the study are included in the article/Supplementary material; further inquiries can be directed to the corresponding author.

## Supplementary material

Supplementary File 1 PCV2 histopathology, ISH and qPCR results for all analysed lymphoid tissues. The file contains the complete dataset of PCV2 histopathology lesion scoring, visual and digital ISH scoring, and qPCR results. Data include all analysed lymphoid tissues (TO, TBLN, MSLN, and ISLN) from each of the 66 animals, with qPCR results specifically provided for TBLN samples. TO, tonsil; TBLN, tracheobronchial lymph node; MSLN, mesenteric lymph node; ISLN, inguinal superficial lymph node; PCV2, porcine circovirus 2.
